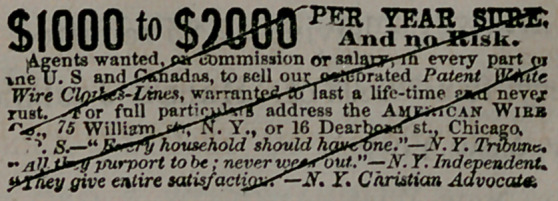# Going Abroad

**Published:** 1869-06

**Authors:** 


### GOING ABROAD.

"Harper's Phrase Book," English, Italian, German and French, being a hand-book for travel, talk for travellers and schools, a guide to conversation in these languages on a new and improved method, intended to accompany

HARPER'S HAND-BOOK FOR TRAVELLERS,

G. W. Pembroke Fettridge, author of "Harper's Hand-Book," assisted by Professors of Heidelberg University, with concise and explicit rules for the pronunciation of the different languages. Sold by Harper & Brothers, New York; by Galignani & Co., 224 Rue Rivoli, Paris; Samson Low & Son and W. S. Adams, 59 Fleet st., London. 75 cents.

Also in flexible morocco cover "Harper's Hand-Book" for

TRAVELLERS IN EUROPE AND THE EAST, being a guide through Great Britain, Ireland, France, Belgium, Holland, Germany, Italy, Egypt, Syria, Turkey, Greece, Switzerland, Tyrol, Spain, Russia, Denmark and Sweden; with a railroad map, corrected up to 1869, sold at the same places with Harper's Phrase Book; it contains a list also of all of our ministers abroad; our consuls, the places where money is paid on letters of credit; with tables of the value of the coins of various countries; hotels, boarding houses, prices of fare, railroads, steamboats, stages, steamships, &c., &c. Price $7.50. This book is an invaluable help, and important to every traveller, as we have formerly experienced; it is exact, truthful, and reliable on all points. This book is corrected annually, while most European guides are from two to ten years old.

LEE & SHEPARD, of Boston, Mass., have published a number of valuable, interesting and instructive books in attractive binding, clear large type and white paper, and their prices will be always found reasonable.

Juliette, or, Now and Forever. By Mrs. Madeline Leslie, pp. 416, 12mo.

General Grant, Our Standard Bearer, his Youth, Manhood, Campaigns, &c., as seen and related by Captain Bernard Galligesken, cosmopolitan, and written out by Oliver Optic and illustrated by Thomas Nast. pp. 348.

Sydine Adriance, or, Trying the World. By Amanda M. Douglas, author of "In Trust," "Stephen Dane," "Claudine."

Grant and Colfax, People's edition, being the life and public services of Gen. U. S. Grant, from his boyhood to his election as President of the United States, with a biographical sketch of the Hon. Schuyler Colfax, by Charles A. Phelps, late speaker of the Massachusetts House of Representatives, and President of the Massachusetts Senate, embellished with two admirable steel portraits and four illustrations, from designs by Hammatt Billings. pp. 344.

"A Thousand Miles Walk" across the Pampas and Andes of South America, by Nathaniel H. Bishop, with an introduction, by Edward A. Samuels, Esq., author of Ornithology and Oology of New England, &c., &c. pp. 310.

Elm Island Stories, by the Rev. Elijah Kellogg, author of "Spartacus the Gladiator," "Good Old Times," &c., with illustrations. pp. 288.

"Twelve Nights" in the Hunter's Camp, being a narrative of real life, illustrated, by G. G. White. pp. 268.

"Salt Water Dick," by May Mannering, pp. 230, being the 5th volume of "The Helping Hand" series, a volume for sailors and those who love the sea.

"Alice's Adventures in Wonderland," by Lewis Carroll, with 42 illustrations by John Tenniel. pp. 192. Beautifully bound and a delight to all the little ones.

"Dotty Dimples' Stories," by Sophie May, author of "Little Prudie Stories," illustrated. pp. 168.

"The True Woman," a series of discourses by Rev. J. D. Fulton, Tremont Temple, Boston, to which is added Woman vs. Ballot, being number nine of Tracts for the People, pp. 264. Sent for 50 cents by mail. The cloth edition is $1.

"The opening sentence is the key-note: 'Three facts stand in the way of woman's being helped by the ballot—God, Nature and Common sense.' Its aim is to set forth the divine ideal for woman in her various relations, and under such heads as Woman as God Made Her, Woman a Helpmeet, the Glory of Motherhood, Woman's Work and Woman's Mission, he presents his views in the pungent, epigrammatic manner characteristic of him either as pastor or author. He believes that woman's mission is different from that of man: that the position assigned to her by the Bible is the only one in which she can attain a symmetrical development, and meet all the obligations devolving upon her. Society owes to her love, honor and protection, and her social and religious rights should be carefully guarded."

Public Baths Free to All, from sun rise to 10 p.m., is an enterprize which commends itself to the co-operation of all humane and benevolent men; the rich are invited to give their money, those who are good and poor are asked for their influence. Address David P. Holton, M.D., 124 West 54th street for circulars, and plans of operation, &c.

VACCINATION. Genuine German Cow Pox Virus, fresh from the cow, at $2 per tube. Free by mail. Also Winton's Vaccinator, silver, $3; gold, $5. Sent free by mail on receipt of price. C. S. Halsey, Pharmaceutist, 147 South Clark st., Chicago, Ill., and 238 Main street, Buffalo, N. Y.

DYSPEPTICS.

HECKER'S WHEATEN GRITS, a highly nutritive, palatable and healthful preparation of wheat grain, invaluable for dyspeptics and for persons of sedentary habits. Two pound papers with directions sold at all the groceries and by Hecker Brothers, 203 Cherry street, N. Y.

The American Tract Society, 150 Nassau st., New York, has issued "The Rescued Child," by Mrs. J. W. Schenck, with illustrations, pp. 96, 16mo. Jane Taylor, pp. 224. By Mrs. Knight; a beautiful and instructive narration for the young; also "THE CRESCENT AND THE CROSS," being a story of the siege of MALTA, by the author of "The Times of Knox and Queen Mary Stuart," &c. 283 pp., in large clear type, both for old and young. It should be remembered that all the publications of this old, useful and honored Society are sold at a very small fraction above cost, the design being not to make money, but to do good by publishing books, which all may read with pleasure and profit both to mind and heart.

WHAT A LADY SAYS

OF MURRAY'S ADVENTURES IN THE WILDERNESS. A beautiful volume of 236 pages is replete, with an interesting account of camp life at the Adirondacks.

"His graphic descriptions of the way they do things in the Wilderness, and the desirable information furnished preparatory to the jaunt, make it a most valuable book for those seeking health. In fact the Adirondacks may favorably compare with what was told us on a visit to Mackinaw more than a quarter of a century ago, viz.: that people never died of disease there, but if they remained until their allotted time come, they would dry up and blow away. Where it can consistently be done, we would recommend having a Parson along, not only for their spiritual good, but to aid them in all rational enjoyments, and to excite their risible faculties, which so much conduce to health. Should they be so fortunate as to secure one of the Murray *Stamp*, they are made up."

ILLUSTRATED BIBLE BIOGRAPHY.

Is one of the most beautiful and solidly useful publications of the American press. To all lovers of the Bible it will be "a well of water springing up" and giving at once satisfaction, instruction and delight, its full title is perhaps the best notice of it, "The Lives and Characters of the Principle Personages, recorded in the Sacred Scriptures with an introduction by

REV. HENRY WARD BEECHER, and an appendix containing the thirty dissertations of the evidences of divine revelation, being a complete summary of Biblical knowledge, carefully condensed and compiled from Scott, Doddridge, Gill, Patrick, Adam Clark, Poole, Lowth, Horne, Wall, Stowe, Robinson, and other eminent writers on the Scriptures, embellished with twenty full paged illustrations, by Gustave Doré, the greatest of living designers, and a presentation page, by Thomas Nast, the celebrated American artist; in addition to which upwards of two hundred and fifty engravings, many of them from celebrated paintings in the principal European art galleries, by such eminent artists as Raphael, M. Angelo, Meyer, Rubens, Salvator Rosa, West, Leborde, Le Suer, De Le Hese, &c., illustrative of scripture scenes, manners and customs, sold only by subscription, Boston, Mass. Published by Lee & Shepard, for W. L. Goss & Co., No. 68 Cornhill. 491 pages, 8vo. Next to the Bible, the books which exemplify and illustrate its meaning by actual historical facts, are of the greatest value, and this volume, so elegantly got up, is one of them, and which every intelligent Christian will consider it a privilege to own.

The May number of THE AMERICAN BUILDER and JOURNAL OF ART, published in Chicago, by Charles D. Lakey, contains a variety of interesting matter pertaining to the building arts, and presents a creditable appearance in its full page illustrations of the First National Bank Buildings, Potter Palmer's Block, and the new Tribune Building. The more noteworthy articles are, "Architecture and Landscape Gardening," "Mortar," "Domestic Economy of Architecture," "The Preservation of Timber," with able editorials on "Real Estate," "Worthless Wooden Pavements," and the "Raid on Western Railways." The design for a Country Villa, with specifications is valuable. THE BUILDER is emphatically a builder's paper, and is worth its price, $3 per annum.

Frank Leslie's Illust'd Newspaper. Weekly, 10c., or $4 a year.
Frank Leslie's Chimney Corner. Weekly, 10c., or $4 a year.
Frank Leslie's Illustrirte Zeitung. Weekly, 10c., or $4 a year.
Frank Leslie's New World. Weekly 6 Cts., or $3 a year.
Frank Leslie's Boys' and Girls' Weekly. 5c., or $2.50 a year.
Frank Leslie's Budget of Fun. 12 Cts., or $1.50 a year.
Frank Leslie's Lady's Magazine and "Gazette of Fashion," the best Magazine published monthly, $3.50 a year.
Frank Leslie's Young Lady's Budget of Fashion, monthly, 20 Cts., or $2 a year.
Frank Leslie's Pleasant Hours. Monthly, 15c., or $1.50 a year.
Frank Leslie's Illustrated Almanac, 30 Cts.
Frank Leslie's Lady's Almanac, 20 Cts.
Frank Leslie's Comic Almanac, 15 Cts.

FRANK LESLIE, 537 Pearl Street, N. Y.

COMPANION TO THE BIBLE. By Rev. E. P. Barrows, D.D., Professor of Biblical Literature, 639 pp. Published by the American Tract Society, 150 Nassau st., N. Y. It is a standard publication of that valuable class of books, which are intended to illustrate the meaning of the Holy Scriptures and to strengthen our faith in their divine origin. It is just such a book as ought to be read by every intelligent Christian.

THE BOOK FOR TRAVELLERS.

Nelson & Sons, 137 Grand st., N. Y., have issued one of the most interesting books of travel lately published, "The Giant Cities of Bashan." 12mo, 377 pp., large print, clear type, and attractive binding, containing a description of "Syria's Holy Places," by the Rev. J. L. Potter, A.M., author of "Five Years in Damascus," Murray's Hand-Book for Syria and Palestine," "The Pentateuch and the Gospels," &c., &c., with instructive illustrations of several hundred texts of Scripture, a general table of contents and a copious index. To those who hope to travel, who have travelled, to clergymen, to all Christian people and intelligent minds of every class this book is one of surprising interest and instruction.

THE MOTHER AT HOME. $2 a year, is a monthly edited by Mrs. Henry Ward Beecher, whom address at 82 Columbia st., Brooklyn. The magazine itself is published at 56 Cedar st., New York, opposite the Post Office, when we have seen several numbers; we will speak of it again.

The Manufacturer and Builder, $1.50 a year, single copies 15 cents. Is published by Western & Co., 37 Park Row, New York; it is in quarto form and gives a large amount of information of practical value to that large class of our most useful citizens from whose occupation it takes its name; its agents are the American News Co., 121 Nassau st., N. Y.

THE APPETITE FOR TOBACCO DESTROYED.—Leave off chewing and smoking the poisonous weed, Tobacco. ORTON'S PREPARATION established 1866. One box of Orton's Preparation is warranted to destroy the appetite for Tobacco in any person, no matter how strong the habit may be. If it fails in any case, the money will be refunded. It is perfectly safe and harmless in all cases. It is almost impossible to break off the use of Tobacco by the mere exercise of the will. Something is needed to assist nature in overcoming a habit so firmly rooted. With the help of the Preparation, there is not the least trouble. Hundreds have used it who are willing to bear witness to the fact that Orton's Preparation completely destroys the appetite for Tobacco, and leaves the person as free from any desire for it as before he first commenced its use. The Preparation acts directly upon the same glands and secretions affected by Tobacco, and through these upon the blood, thoroughly cleaning the poison of Tobacco from the system, and thus allaying the unnatural cravings for Tobacco. No more hankering for Tobacco after using Orton's Preparation! Recollect, it is warranted. The time taken to allay all desire for Tobacco, by the use of the Preparation, varies slightly in different persons—the average time being about five days. Some have no desire for Tobacco whatever after using the Preparation two days.

The Health and Purse of every Tobacco user in the country calls loudly, Abandon the use of Tobacco!

*Recommendations.*—The following are a few selected from the multitude of recommendations in our possession:

We, the undersigned, hereby certify that we have used Orton's Preparation for the purpose of destroying the appetite for Tobacco, and can assure those who are suffering from this habit that Orton's Preparation will certainly destroy the appetite for Tobacco quickly and permanently, and without any bad effect upon the health, and without creating an appetite either for the Preparation or any substitute.

W. P. Heald, Bangor, Maine; J. Moody, Southport, Indiana; E. W. Adkins, Knoxville, Tenn.; John Merrill, Bangor, Maine; J. Bunch, Springfield, Tenn.; W. D. Harrington, West Point, Ga.

*From Samuel Cassidy, Editor of the "Journal and Argus."* PETALUMA, (Cal.,) December 12, 1868.—For about twenty years I had used Tobacco in its various forms, and for the past eight years had been an inveterate smoker. Becoming satisfied that the excessive use of this narcotic was seriously affecting my health, I determined, if possible, to break myself of the habit. Hearing of Orton's Preparation for destroying the appetite for Tobacco, I sent to Portland, Maine, for a box of the medicine, which I received through the mail on the 13th of November. A month has not elapsed, and yet the medicine has effectually relieved me from every craving or desire to use Tobacco in any form. The Preparation is not more unpleasant to take than common chewing gum. I conscientiously believe the Preparation will have the promised and desired effect in every instance where it is given a fair trial. Upon that belief, and from an honest desire to assist others who wish to break away from the slavish appetite for Tobacco, I offer this testimonial.—SAMUEL CASSIDY.

☞ Beware of counterfeits and all articles purporting to be like his, of the same name or otherwise. The great popularity of Orton's Preparation has induced unprincipled persons to attempt palming upon the public counterfeit and inferior articles. Purchasers will please order directly from the proprietor, or his duly authorized agents. THE PRICE of Orton's Preparation is $2 per box, or three boxes for $5, sent by mail to any part of the country, securely sealed from observation, with passage paid, on receipt of price. How to send money by mail: Inclose the amount in a letter, seal carefully, register your letter, and take a receipt for it from your Postmaster. Money sent by mail as above directed at my risk. An agent wanted in every town. Address C. B. COTTON, Proprietor, Box 1,748, Portland, Maine.

THE LONDON LANCET is published monthly, simultaneously with the London issue, by the energy and enterprize of Wm. C. Herald, No. 32 Beekman Street, New York, at $5 a year; postage 24 cts per annum; supplied to the trade by the American News Co. at 121 Nassau Street, New York. The high character of the Lancet, as "authority," by the medical profession of the United States and Canada, is well understood, and it is well worth the patronage of practising physicians, young and old, as it enables them to keep up with the advanced opinions, improvements, and discoveries of the times, both in surgery and medicine.

WM. WOOD & CO. have published "ANAL FISSURE, its Aetiology, Pathology, Diagnosis and Treatment," by William Bodenhamer, A.M. M.D., author of Congenital Diseases of the Rectum, and Professor of Diseases of the Rectum and Contiguous Textures, 199 pp. 8vo., illustrated by numerous cases and drawings. The editor, after a personal acquaintance with the Author of more than thirty years, and a familiarity with his mode of practice, does not hesitate to pronounce Dr. Bodenhamer as the most successful living practitioner at home or abroad in ANAL FISSURE, FISTULA, STRICTURE of the bowels, PILES &c.; never using caustic or the knife, but with manipulations performed in a manner peculiar to himself, exhibited and communicated to the Profession with great courtesy and cheerfulness when called upon at his residence, 237 5th Ave., New York; he has not only obtained its confidence and respect here, but has drawn to himself an amount of practice from a list of patients of which he may well be proud, members of various state legislatures, governors, senators, officers in the Army and Navy, and from all the professions, and we feel that we are doing our readers a very great service in making these statements, (without the author's knowledge) in case any of them should need his skill in the direction referred to.

As the cases given are very instructive to the general reader, and the causes of these most distressing diseases are plainly stated, and by knowing them the maladies may be avoided, we will keep the book for sale in our office, or will send it post-paid on having $2.25 remitted in a post office order, for safety, to address of HALL'S JOURNAL OF HEALTH, 176 Broadway, New York.

## Figures and Tables

**Figure f1:**